# Acceptable symbiont cell size differs among cnidarian species and may limit symbiont diversity

**DOI:** 10.1038/ismej.2017.17

**Published:** 2017-03-21

**Authors:** Elise Biquand, Nami Okubo, Yusuke Aihara, Vivien Rolland, David C Hayward, Masayuki Hatta, Jun Minagawa, Tadashi Maruyama, Shunichi Takahashi

**Affiliations:** 1 Plant Science Division, Research School of Biology, The Australian National University, Canberra, Australian Capital Territory, Australia; 2 Department of Economics, Tokyo Keizai University, Kokubunji, Tokyo, Japan; 3 Division of Environmental Photobiology, National Institute for Basic Biology, Okazaki, Japan; 4 Australian Research Council Centre of Excellence for Translational Photosynthesis, Plant Science Division, Research School of Biology, The Australian National University, Canberra, Australian Capital Territory, Australia; 5 Graduate School of Humanities and Sciences, Ochanomizu University, Tokyo, Japan; 6 Department of Basic Biology, School of Life Science, SOKENDAI (The Graduate University for Advanced Studies), Okazaki, Japan; 7 Japan Agency for Marine-Earth Science and Technology (JAMSTEC), Yokosuka, Kanagawa, Japan

## Abstract

Reef-building corals form symbiotic relationships with dinoflagellates of the genus *Symbiodinium*. *Symbiodinium* are genetically and physiologically diverse, and corals may be able to adapt to different environments by altering their dominant *Symbiodinium* phylotype. Notably, each coral species associates only with specific *Symbiodinium* phylotypes, and consequently the diversity of symbionts available to the host is limited by the species specificity. Currently, it is widely presumed that species specificity is determined by the combination of cell-surface molecules on the host and symbiont. Here we show experimental evidence supporting a new model to explain at least part of the specificity in coral–*Symbiodinium* symbiosis. Using the laboratory model *Aiptasia*–*Symbiodinium* system, we found that symbiont infectivity is related to cell size; larger *Symbiodinium* phylotypes are less likely to establish a symbiotic relationship with the host *Aiptasia*. This size dependency is further supported by experiments where symbionts were replaced by artificial fluorescent microspheres. Finally, experiments using two different coral species demonstrate that our size-dependent-infection model can be expanded to coral–*Symbiodinium* symbiosis, with the acceptability of large-sized *Symbiodinium* phylotypes differing between two coral species. Thus the selectivity of the host for symbiont cell size can affect the diversity of symbionts in corals.

## Introduction

Endosymbiotic dinoflagellates of the genus *Symbiodinium* reside in many cnidarian organisms, such as reef-building coral, sea anemone, jellyfish and hydrocoral (Davy *et al.*, 2012). The *Symbiodinium*–cnidarian association is a mutualistic symbiotic relationship, with each member supporting the other to survive in an oligotrophic environment. *Symbiodinium* provide photosynthetically fixed carbon and in return receive inorganic nutrients from the host cnidarian (Yellowlees *et al.*, 2008). *Symbiodinium* is a key primary producer of coral reefs and the coral–*Symbiodinium* symbiotic relationship is a cornerstone for biologically diverse coral reef ecosystems.

The first step in initiating a symbiotic relationship is recruiting *Symbiodinium* into the cnidarian host’s cells. There are two main ways for this to occur; they can be inherited from the parent (vertical transmission) or obtained directly from the environment (horizontal transmission). Vertical transmission occurs in select host taxa (for example, brooding corals) that release eggs or brooded planula larvae only after symbionts have been transferred to the offspring. Horizontal transmission could potentially happen in any host taxon, mainly in the early developing stages but can also occur in the adult stage (Baker, 2003).

Recent molecular and genetic analysis classified *Symbiodinium* into nine large groups called clades (A–I) with each clade containing multiple phylotypes (Pochon and Gates, 2010). Between *Symbiodinium* phylotypes, morphological differences can be seen in chromosome number, cell size of both the vegetative and motile phases and in the chloroplast number, size and arrangement (Trench, 1979; LaJeunesse, 2001). This variation leads to physiological differences among phylotypes, including their sensitivity to environmental stresses such as increased seawater temperature or strong light (Tchernov *et al.*, 2004; Takahashi *et al.*, 2008; Takahashi *et al.*, 2013). Hosts can harbor multiple *Symbiodinium* phylotypes and the dominant *Symbiodinium* phylotype can vary with changes in the environmental conditions. For the host to survive and adapt to changing conditions, it can be critical that it harbors *Symbiodinium* phylotypes that are suitable to the new environment (Baker, 2001; Baker *et al.*, 2004; Lewis and Coffroth, 2004; Rowan, 2004; Berkelmans and van Oppen, 2006; Jones *et al.*, 2008; Howells *et al.*, 2012).

The uptake of *Symbiodinium* from the environment into host cells is restricted by species specificity. Laboratory infection tests using sea fans (Kinzie, 1974), upside-down jellyfish (Fitt, 1985), sea anemone (Schoenberg and Trench, 1980; Belda-Baillie *et al.*, 2002; Rodriguez-Lanetty *et al.*, 2003; Hambleton *et al.*, 2014) and coral (Weis *et al.*, 2001) demonstrated that the host could only establish a symbiotic relationship with specific *Symbiodinium* phylotypes. Furthermore, the degree of specificity differed among both the host species and the *Symbiodinium* phylotypes (LaJeunesse, 2002; Baker, 2003), restricting the range of compatible partners. In corals, the *in situ* symbionts can differ between larvae and adult polyps (Little *et al.*, 2004). Therefore, it has been suggested that species specificity can vary with life stage. However, a study in the sea anemone *Aiptasia* showed that symbiont specificity did not change with life stage (Hambleton *et al.*, 2014).

In experiments looking at the symbioses between green *Hydra* and *Chlorella* (Meints and Pardy, 1980) or marine cnidarians and *Symbiodinium* (Lin *et al.*, 2000; Wood-Charlson *et al.*, 2006), the pattern recognition receptors on the host cell membrane and the microbe-associated molecular patterns on the symbiont cell surface were proposed to be important in initiating the symbiotic relationship (Davy *et al.*, 2012). It was found that molecular patterns, that is, glycans, differ among *Symbiodinium* strains (Wood-Charlson *et al.*, 2006; Logan *et al.*, 2010), suggesting that the combination of cell-surface molecules on the host and symbiont could mediate species specificity. However, direct experimental data supporting this hypothesis is still lacking, and the mechanisms associated with cnidarian–algae specificity are still poorly understood.

In this study, we investigated the relationship between infectivity and *Symbiodinium* cell size using two different types of host: artificially bleached sea anemone (*Aiptasia* sp.) and aposymbiotic juvenile polyps of two different coral species (*Acropora tenuis* and *Cyphastrea serailia*). Our results demonstrated that cell size affected the infectivity of *Symbiodinium* phylotypes and that the maximum threshold for symbiont cell size differed among coral species.

## Materials and methods

### Cultures and growth conditions

All *Symbiodinium* strains used for infection tests were obtained from the National Center for Marine Algae and Microbiota (East Boothbay, ME, USA), CSIRO Australian National Algae Culture Collection (Hobart, TAS, Australia) or the Buffalo Undersea Reef Research Culture Collection (Buffalo, NY, USA). To ensure that cultures were monoclonal, all *Symbiodinium* phylotypes were subcultured from a single cell isolated by a cell sorter (BD FACS Aria II, BD Biosciences, San Jose, CA, USA). *Symbiodinium* strains used in this study did not always correspond to the information provided by the culture collection centers, that is, genotype (subclade) of *Symbiodinium* phylotypes (Supplementary Table S1). To avoid confusion, *Symbiodinium* phylotypes whose genotype did not match the provided information were renamed (Supplementary Table S1). All *Symbiodinium* strains used in Figure 6 to examine the relationship between clade and cell size were obtained from Buffalo Undersea Reef Research Culture Collection and categorized according to the genotype information provided by the culture collection; they are all expected to be monoclonal lines.

*Symbiodinium* cells were grown in filtered (0.22 μm pore filter; Steritop-GP Filter Unit, Merck Millipore, Billerica, MA, USA) artificial seawater (sea salt; Sigma-Aldrich, St Louis, MO, USA) containing Daigo’s IMK medium for marine microalgae (Wako, Osaka, Japan). *Symbiodinium* cells were grown at a continuous temperature of 23 °C under photosynthetically active radiation at 80 μmol photons m^−2^ s^−1^ with 16 h light (white color, fluorescence tubes) in a day. *Symbiodinium* cells that had reached the mid-logarithmic phase of growth were harvested by centrifugation at 2000 *g* for 3 min and resuspended in fresh seawater media.

The sea-anemone used in our study is *Aiptasia* (cf. insignis) isolated from Okinawa (Japan) >10 years ago (Belda-Baillie *et al.*, 2002). This *Aiptasia* originally harbored a clade B *Symbiodinium* (GenBank accession number AF289267; Belda-Baillie *et al.*, 2002). *Aiptasia* polyps used in our study were monoclonal, originating from a single polyp. *Aiptasia* were cultured in the presence of a mixture of *Symbiodinium* phylotypes from clades A and B. *Aiptasia* were grown under the same light and temperature conditions as *Symbiodinium* using artificial seawater without Daigo’s IMK. Before infection, *Aiptasia* were artificially bleached (aposymbiotic *Aiptasia*) by incubating them at 33 °C in complete darkness for 3 weeks and then returned to normal growth temperature for >3 weeks. Seawater was changed every 2 days during bleaching treatment. Bleaching was confirmed by the inability to detect any symbiotic algae under a MZ FLIII fluorescence stereomicroscope (Leica Microsystems, Heerbrugg, Switzerland). Once *Aiptasia* polyps were bleached, they were cultured separately from other *Aiptasia* polyps, in the absence of *Symbiodinium*. Freshly hatched *Artemia nauplii* were fed to *Aiptasia* once a week.

*A. tenuis* egg–sperm bundles were collected from several colonies near the Sesoko marine station, Okinawa, Japan (University of the Ryukyus) in June 2013. *C. serailia* egg–sperm bundles were collected from several colonies at the Seto Marine Biological Laboratory, Wakayama, Japan (Kyoto University) in July 2013. Bundles collected from several colonies were combined and incubated for a few hours to allow fertilization to occur. Embryos were then transferred to fresh seawater that had been filtered through a 3 μm polypropylene cartridge (TCW-3N-PPS, Advantec, Tokyo, Japan) with a filter housing (1PA, Advantec) or a 0.22 μm filter (Steritop-GP Filter Unit, Merck Millipore). Planula larvae were maintained in filtered seawater at 25 °C until use. Metamorphosis was induced in *A. tenui*s with 1 μM Hym-248 (Iwao *et al.*, 2002), and juvenile polyps were distributed into six-well plates. For *C. serailia*, planula larvae were kept in polystyrene boxes as the juvenile polyps did not settle in plastic petri dishes. Peptide treatment, such as Hym-248, was not necessary to induce metamorphosis in *C. serailia*. All juvenile polyps were cultured in filtered artificial seawater until use.

### Measurement of Symbiodinium cell size

The size of *Symbiodinium* cells was measured with an automated cell counter (Cellometer Auto X4; Nexcelom Bioscience, Lawrence, MA, USA). *Symbiodinium* cells were collected in the middle of the light period by centrifugation (16 000 *g*) for 1 min. The collected *Symbiodinium* cells were resuspended in fresh seawater and mixed by vortexing. After these steps, *Symbiodinium* cells lost their flagella and were all in the vegetative form (round cell shape without flagella). Measurements were conducted three times using separately cultured cells. Motile and vegetative *Symbiodinium* cells (Supplementary Figure S1) were photographed using a Leica DM6000B microscope (Leica Microsystems) equipped with a SPOT Flex camera (SPOT Imaging Solutions, Sterling Heights, MI, USA).

### Infection of Aiptasia by Symbiodinium

To examine the infectivity of *Symbiodinium* into *Aiptasia*, single aposymbiotic polyps were placed in multiple clear plastic cups with 30 ml of artificial seawater and each mixed with different *Symbiodinium* strains (40 000 cells ml^−1^). The number of *Symbiodinium* cells was measured with an automated cell counter (TC20, Bio-Rad, Hercules, CA, USA). Three *Aiptasia* polyps cultured in separate petri dishes were used for each *Symbiodinium* strain. Infection of *Aiptasia* by *Symbiodinium* was confirmed by monitoring symbiont chlorophyll fluorescence in tentacles using a MZ FLIII fluorescence stereomicroscope (Leica Microsystems) with a GFP2 filter (excitation: 480/40, emission: 510LP) and a digital camera (EOS 600D, Canon, Tokyo, Japan). Infection of *Aiptasia* polyps was evaluated on days 1, 2, 3, 4, 6, 8, 9, 10, 11, 14, 16, 18, 21, 23, 25 and 35 after mixing with *Symbiodinium*. Infection was determined by examination in a separate container with fresh seawater to avoid counting the *Symbiodinium* cells in the media. *Aiptasia* polyps were then returned to the original seawater containing *Symbiodinium* cells. The *Aiptasia* polyp was said to be infected when >30 foci of *Symbiodinium* cells could be seen within a tentacle.

### Incorporation of fluorescent microspheres into Aiptasia

Fluoresbrite carboxylate yellow-green microspheres (2.5% solids-latex, excitation max.=441 nm; emission max.=486 nm) with diameters of 6.3±0.18, 10.4±0.25 and 11.4±0.30 μm (Polysciences, Inc., Warrington, PA, USA) were used to examine the uptake of microspheres into host cells. The 11.4 μm microspheres were custom made. The size of microspheres was verified with an automated cell counter (Cellometer Auto X4). Each suspension of microspheres (~12 000 microspheres) was mixed with 30 fresh *A. nauplii* and the mixed pellet (approximately 1 mm in diameter) was fed using forceps to individual *Aiptasia* polyps harboring *Symbiodinium* (that is, *Aiptasia* polyps cultured in the presence of a mixture of *Symbiodinium* phylotypes from clades A and B). Microspheres could be taken into the host cells without mixing with *Artemia*. However, mixing with *Artemia* was necessary to ensure that the same number of microspheres was introduced into the stomach in each experiment. Three *Aiptasia* polyps were used for each microsphere size. The number of microspheres was counted with a TC20 automated cell counter. Incorporation of microspheres into *Aiptasia* tentacles was confirmed by imaging yellow-green fluorescence using a fluorescence stereomicroscope (see Infection of *Aiptasia* by *Symbiodinium* section). Incorporation was evaluated after 6 h, 1 day, 2, 3 and 6 days. The *Aiptasia* polyp was said to have incorporated microspheres when >30 foci of microspheres could be seen within a tentacle.

To confirm that the microspheres were inside host cells, we examined their localization by laser-scanning confocal microscopy. Fluoresbrite carboxylate yellow-green microspheres (6.3 μm in diameter) were fed to *Aiptasia* polyps as described above. After 3 days, *Aiptasia* polyps were incubated in sea water supplemented with 190 mM MgCl_2_ to stop their movement and then fixed with 10% formalin for 1 h. Fixed *Aiptasia* polyps were washed with seawater and placed on 0.5% agarose gel on a glass depression slide and covered with a glass cover slip. Images of *Aiptasia* tentacles were taken using a LSM780 laser-scanning confocal microscope (Zeiss, Oberkochen, Germany) equipped with a 40 × objective (NA=1.1). Fluoresbrite microspheres and *Symbiodinium* were excited at 488 nm (emission: 499–561 nm) and 633 nm (emission: 648–735 nm), respectively. Transmitted light was collected after excitation at 633 nm. *Z*-stacks were taken (0.7 μm between each single plane) and processing, including 3D reconstruction and depth indexation, was performed using the Zen 2012 software package (Zeiss).

### Infection of corals by Symbiodinium

To examine the infectivity of *Symbiodinium* into corals, aposymbiotic polyps were mixed with different *Symbiodinium* strains (40 000 cells ml^−1^). The number of *Symbiodinium* cells was measured with a Thoma hemocytometer (SLGC, Tokyo, Japan). Infection of coral polyps by *Symbiodinium* was evaluated by examining *Symbiodinium* cells (brownish color) using stereomicroscope (Leica M165C) fitted with a digital camera (Leica MC120HD). Examinations were carried out in fresh seawater to avoid counting the *Symbiodinium* cells in the media. Polyps were then replaced into the original seawater containing *Symbiodinium* cells. Polyps were replaced in fresh seawater media containing the *Symbiodinium* strains (40 000 cells ml^−1^) every 2 days.

### Symbiodinium genotyping

Total DNA was isolated from approximately 5 × 10^5^ cultured *Symbiodinium* cells using the DNeasy Plant MiniKit (Qiagen, Hilden, Germany). The complete internal transcribed spacer 2 (ITS2) region was amplified using Platinum Taq DNA Polymerase High fidelity (Invitrogen, Carlsbad, CA, USA) using the primers ITSintfor2 (LaJeunesse, 2002) and ITS2rev (Apprill and Gates, 2007). PCR conditions were as described in Apprill and Gates (2007). Amplified products were purified with the QIAquick PCR Purification Kit (Qiagen) and ligated into pGEM-T Easy (Promega, Madison, WI, USA). Plasmids containing inserts were isolated using the QIAGEN Plasmid Mini Kit (Qiagen) and amplified using illustra TempliPhi (GE Healthcare, Piscataway, NJ, USA). DNA was sequenced (6–20 clones per culture) using Big Dye Terminator v. 3.1 (Applied Biosystems, Foster City, CA, USA) and reactions were run on an ABI 3730 sequencer at the Biomolecular Resource Facility (John Curtin School of Medical Research, Australian National University, Acton, ACT, Australia). Sequences were analyzed using DNASTAR (Lasergene, Madison, WI, USA) and MacVector (Accelrys, San Diego, CA, USA) and assigned to clades by performing BLAST searches against the ITS2 sequence database from GeoSymbio (Franklin *et al.*, 2012) or GenBank.

### Statistical analysis

**Figure 1:**
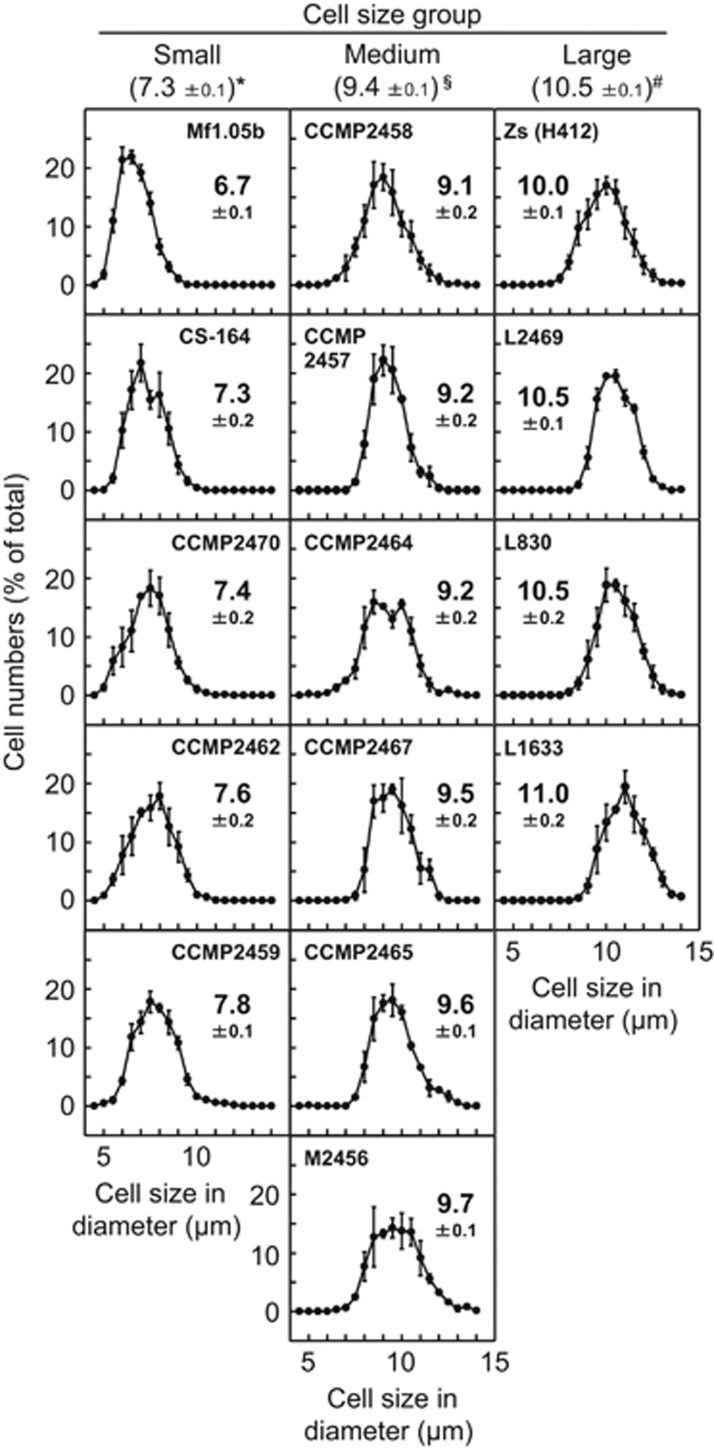
Distribution of cell size in cultured *Symbiodinium* strains. The diameter of individual cells of each strain was measured with an automated cell counter (>100 cells in each measurement) and plotted as a percentage of the total sampled population. Numbers besides the plot in each panel show the average cell diameter (μm)±s.e. (*n*=3). Numbers below the *Symbiodinium* cell size group headings show the average cell diameter (μm)±s.e. for all strains in that group. Different symbols (*, § and #) indicate significant difference (*P*<0.01) between groups.

**Figure 2:**
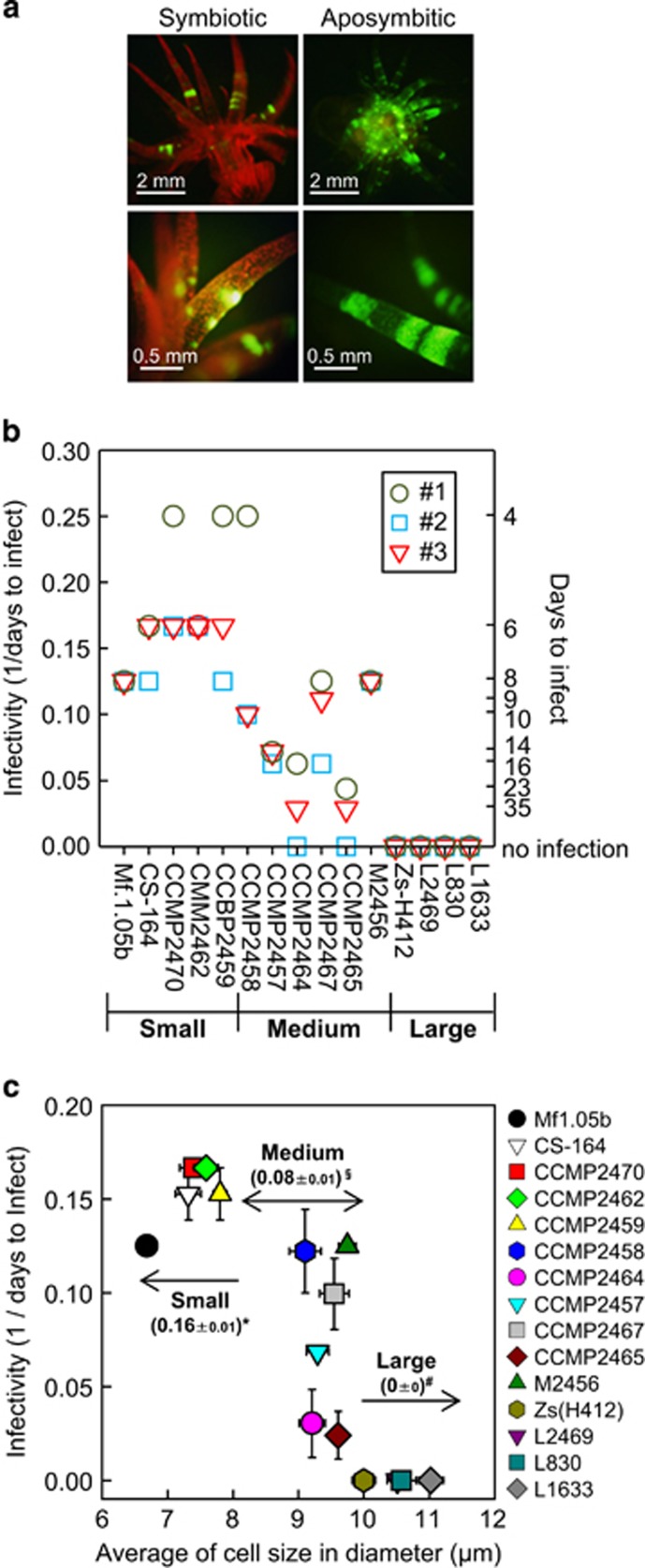
Infection of different *Symbiodinium* strains into *Aiptasia*. (**a**) Fluorescence stereomicroscope images of *Aiptasia* anemones with (symbiont) and without (aposymbiont) symbiotic algae *Symbiodinium*. Red fluorescence is from the chlorophyll in *Symbiodinium*, while the autofluorescence of *Aiptasia* is green. (**b**) Days to infect (the number of days until infection was verified) and infectivity (1/days to infect) are shown on the right and left *y* axes, respectively. An *Aiptasia* polyp was said to be infected when >30 foci of *Symbiodinium* cells could be seen within a tentacle. Three independent experiments (#1, #2 and #3) were carried out in each *Symbiodinium* strain. (**c**) Relationship between *Symbiodinium* cell size and infectivity. Numbers in the panel show the average infectivity (1/days to infect)±s.e. of each size group. Different symbols (*, § and #) indicate significant difference (*P*<0.01) between groups. Error bars, ±s.e. (*n*=3).

The data for *Symbiodinium* cell size (Figure 1) was analyzed by one-way analysis of variance between the three size groups after the normality was confirmed by Chi-Square Goodness-of-Fit test and the homoscedasticity by Bartlett’s test. When a significant difference was established, Scheffe’s test was employed to determine which group(s) differed. The data of symbiont infectivity (Figure 2c) and symbiont cell size in corals (Supplementary Figure S4) were analyzed between the three size groups using Kruskal–Wallis test. Where a significant difference was established, Steel–Dwass test was employed to determine which group(s) differed.

## Results

### Large-sized Symbiodinium strains failed to infect Aiptasia

We used cultured *Symbiodinium* strains isolated from different host taxa, including scleractinian coral, sea anemone, octocoral, jellyfish, zoanthid and giant clams (Supplementary Table S1). *Symbiodinium* cell size (diameter) was measured using an automatic cell counter. As cell division generally occurs during the dark period (Fitt and Trench, 1983), cell size measurements were conducted in the middle of the light period to avoid counting dividing cells. The average *Symbiodinium* cell size differed among strains and ranged from 6.7 to 11.0 μm (Figure 1). The maximum and minimum cell sizes also differed among strains but scaled with their average cell size, that is, the maximum and minimum cell sizes were smaller in the smaller *Symbiodinium* strains (Figure 1). In this study, we classified *Symbiodinium* strains into three groups based on their average cell diameter: small (<8.0 μm, *n*=5), medium (8.0–10.0 μm, *n*=6), and large (>10.0 μm, *n*=4; Figure 1). This cell size classification was consistent for both the motile (with flagella) and vegetative life stages (Supplementary Figure S1).

We tested the infectivity of each *Symbiodinium* strain using artificially bleached aposymbiotic *Aiptasia* anemones. After bleaching, *Aiptasia* lost all *Symbiodinium*-associated red chlorophyll fluorescence and showed only its intrinsic green fluorescence (Figure 2a). To test infection efficiency, an individual *Aiptasia* polyp was separately exposed to a single *Symbiodinium* strain. Infection by *Symbiodinium* was monitored by observing the recovery of chlorophyll fluorescence in the host over 35 days (Supplementary Figure S2). An *Aiptasia* polyp was said to be infected when >30 foci of *Symbiodinium* cells could be seen within an *Aiptasia* tentacle. Infection follows the uptake of *Symbiodinium* cells into the gastrovascular cavity (stomach). All *Symbiodinium* strains were taken into the stomach within 2 days after inoculation. However, infection was achieved by only 11 of the 15 *Symbiodinium* stains tested (Figure 2b). The time taken to infect varied considerably between *Symbiodinium* strains, ranging from 5 to 35 days, consistent with a previous report (Belda-Baillie *et al.*, 2002). Additionally, under our experimental conditions, infection by *Symbiodinium* CCMP2464 and CCMP2465 was very slow and was only achieved in two of the three replicate experiments (Figure 2b). *Aiptasia* incubated under the same experimental conditions without *Symbiodinium* remained bleached after 35 days (Supplementary Figure S2), indicating that there was no *Symbiodinium* contamination in our bleached polyps or in the seawater.

We next examined the relationship between infectivity and *Symbiodinium* cell size (Figure 2c). Infectivity was very high for all small *Symbiodinium* strains while it was lower and considerably more varied for *Symbiodinium* strains of medium size (Figure 2c). Importantly, all of the *Symbiodinium* strains that were unable to infect *Aiptasia* were large (>10 μm; Figure 2c). These results suggest that *Symbiodinium* cell size is inversely related to infectivity into *Aiptasia* anemone, that is, the larger the cell size, the lower the infectivity.

### Size-dependent uptake of artificial microspheres into host cells

**Figure 3:**
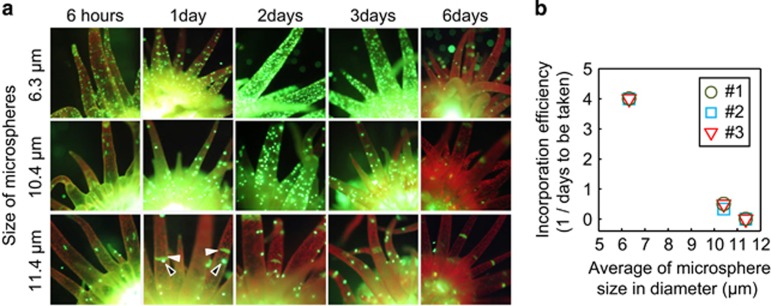
Incorporation of microspheres of different sizes into *Aiptasia*. (**a**) Fluorescence stereomicroscopic images of *Aiptasia* anemones after introducing yellow-green fluorescent microspheres of different sizes (6.3±0.18, 10.4±0.25, and 11.4±0.3 μm in diameter). Microspheres of each size were separately fed into the mouths of anemones and their presence in tentacles was monitored for 6 days. White and black arrow heads indicate autofluorescence of *Aiptasia* and microspheres, respectively. (**b**) Relationship between incorporation efficiency (1/the number of days until incorporation was verified) and average microsphere size. An *Aiptasia* polyp was said to have incorporated microspheres when >30 foci could be seen within a tentacle. Three independent experiments (#1, #2 and #3) were carried out in each size.

To determine whether cell size is a determinant of symbiont specificity in *Aiptasia*, we monitored the incorporation of different-sized yellow-green fluorescent microspheres (6.3±0.18, 10.4±0.25, and 11.4±0.30 μm in diameter) into *Aiptasia* cells (Figure 3). In each experiment, approximately 12 000 microspheres were fed directly into the mouth of individual *Aiptasia* already harboring *Symbiodinium* (*n*=3) and the number of microspheres located in tentacles was monitored for 6 days (Figures 3a and b). An individual *Aiptasia* polyp was said to have incorporated the microspheres when >30 could be seen within a tentacle. All three *Aiptasia* polyps tested successfully incorporated 6.3 and 10.4 μm microspheres in 6 h and 2–3 days, respectively. Compared with our infectivity assay results, the microspheres were taken up much faster than the *Symbiodinium* cells. This is possibly because the microspheres were fed directly into the mouth of the host and were therefore quickly and abundantly taken up into the gastrovascular cavity. Importantly, none of the *Aiptasia* polyps tested with the 11.4 μm microspheres reached the threshold needed to ensure that they had incorporated the microspheres, although a small number were seen in the tentacles (Figure 3a). Interestingly, all microspheres seen in the tentacles were completely expelled from the polyps after 6 days (Figure 3a). This result indicates that microspheres are not able to be retained in host cells.

**Figure 4:**
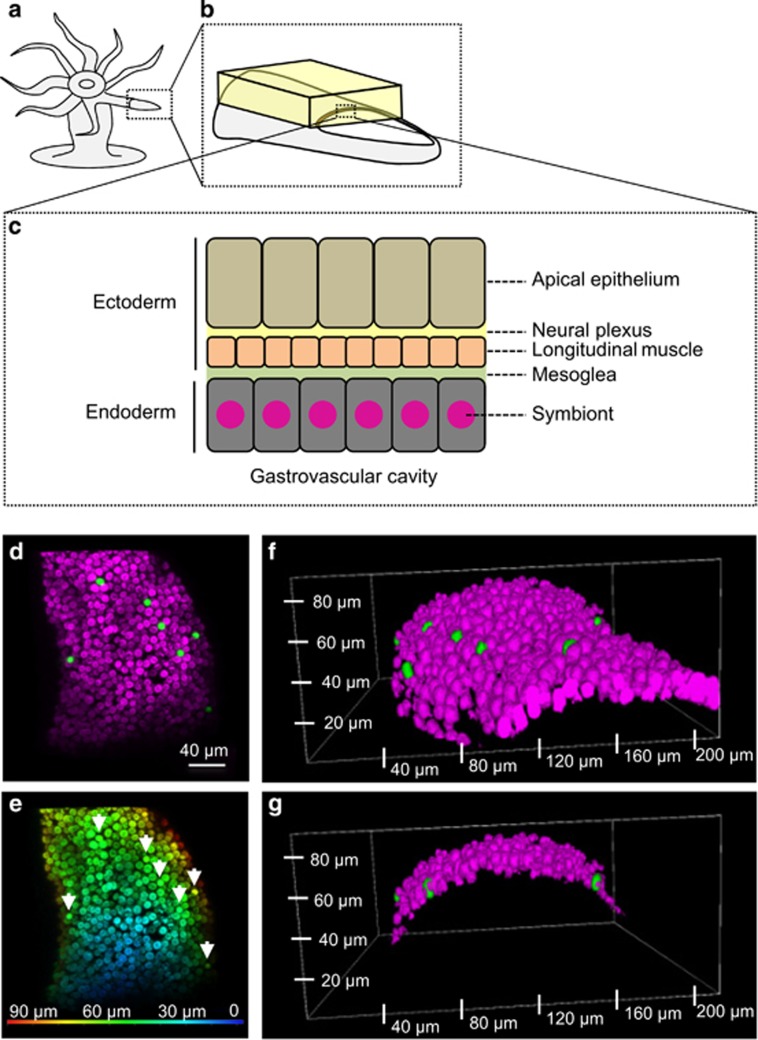
Incorporation of microspheres into *Aiptasia* cells. (**a**–**c**) Illustration of *Aiptasia* sample used. (**b**) The yellow cuboid highlights the volume of tentacle imaged in subsequent panels. (**d**–**g**) Confocal microscopic images of an *Aiptasia* tentacle containing *Symbiodinium* (magenta) and small-sized (6.3±0.18) microspheres (green). (**d**) 3D reconstruction of the cuboid seen from the surface of the tentacle. (**e**) 3D visualization of the same cuboid showing the depth (μm from the surface) of each symbiont or microsphere (arrows). (**f**) 3D reconstruction of the tentacle (side view). (**g**) 3D reconstruction cut through two microspheres (side view).

To determine whether the microspheres seen in the tentacles resided within the host cells or the gastrovascular cavity, we looked at the localization of 6.3 μm microspheres with respect to *Symbiodinium* cells (Figure 4). *Aiptasia* tentacles have a single layer of endoderm cells that face the gastrovascular cavity and harbor the *Symbiodinium* symbionts (Figures 4a–c). In our experiment, the fluorescent microspheres were found in the same layer as the symbiont, interspersed between *Symbiodinium* cells (Figures 4d–g). These results indicated that host endodermal cells were able to take up small microspheres in a similar manner to small *Symbiodinium* with both localizing to the endodermal cell layer, whereas larger microspheres were inefficiently taken into host cells.

### The maximum threshold of symbiont cell size differs among coral species

In our study, all large-sized *Symbiodinium* strains failed to infect *Aiptasia*. These strains were originally isolated from a range of host organisms (Supplementary Table S1), including sea anemones (*Cyphastrea gigantea*, *Aiptasia pallida* and *Aiptasia pulchella*) and zoanthids (*Zoanthus sociatus*) (LaJeunesse, 2001; Santos *et al.*, 2001). However, it cannot be determined whether these *Symbiodinium* strains were residing within host cells or rather were present on the surface of the organisms at the time of isolation. Nevertheless, some coral species (for example, *Pocillopora* sp.) have been shown to harbor large-sized *Symbiodinium* phylotypes (Domotor and Delia, 1986). It is therefore conceivable that the maximum threshold for symbiont cell size differs among host species and that the infectivity of the symbiont is related to both the cell size of the symbiont and the maximum size threshold of the host.

**Figure 5:**
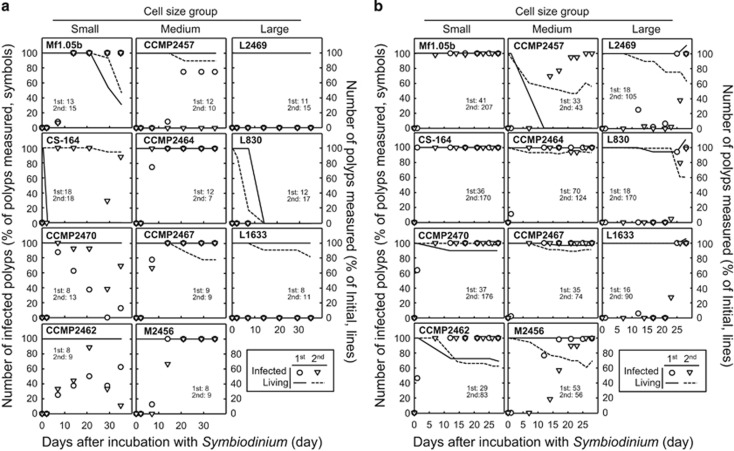
Infection of different *Symbiodinium* strains into corals. Aposymbiotic juvenile polyps of (**a**) *A. tenuis* and (**b**) *C. serailia* were incubated with different *Symbiodinium* strains and monitored for 35 and 30 days, respectively. Number of infected polyps is shown on the left *y* axes (percentage of total polyps measured, symbols). Number of polyps measured at each time point (percentage of initial, lines) is shown on the right *y* axes. Only healthy looking polyps were included in the measurements at each time point. A coral polyp was said to be infected when >30 foci of *Symbiodinium* cells could be seen within a tentacle. Each experiment was carried out twice using two set of polyps in different containers (microplates) and each result is shown separately. First experiment, closed symbols and solids line. Second experiment, open symbols, dashed line. The initial number of polyps used for the first and second experiment is shown in each panel.

To test our hypothesis, we examined the infectivity of 11 *Symbiodinium* strains of different cell size using aposymbiotic juvenile polyps from two coral species; *A. tenuis* and *C. serailia*. These coral species were chosen as they do not undergo vertical transmission and therefore aposymbiont juvenile polyps can be readily obtained in the laboratory from collected egg–sperm bundles. Individual polyps of each coral species were incubated with a single *Symbiodinium* strain and infection was visually monitored over 30 days for *C. serailia* and 35 days for *A. tenuis* (Supplementary Figure S3). In *A. tenuis*, 8 of the 11 *Symbiodinium* strains tested could infect the juvenile polyps (Figure 5a). The three remaining *Symbiodinium* strains were all large sized. Two of these strains failed to infect after 35 days, and the third one (L830) caused all the polyps to die after 14 days (Figure 5a). These results demonstrated that symbiont cell size also influenced infectivity in corals. In *C. serailia*, all *Symbiodinium* strains tested were able to infect the host, including the large-sized strains, albeit their infection being much slower than that of small- and medium-sized strains (Figure 5b). Total infection of all juvenile polyps was achieved in 7 days, 7–25 days, and 25–28 days for small-, medium- and large-sized strains, respectively (Figure 5b). As each *Symbiodinium* culture contained a range of cell sizes (Figure 1), we isolated symbionts from infected *C. serailia* and confirmed the presence of the largest cells of the large-sized *Symbiodinium* strains in the host (Supplementary Figure S4).

### Differences in cell size among Symbiodinium clades

**Figure 6:**
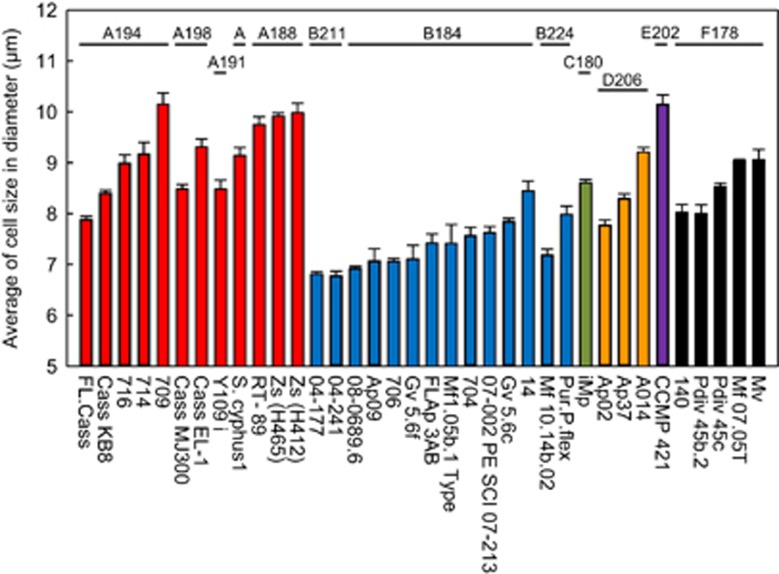
Distribution of *Symbiodinium* cell size among different phylotypes. The average cell size (diameter) of 36 cultured *Symbiodinium* strains was measured with an automated cell counter. Details of the cp23S-rDNA phylotypes (A194, A198, A191, A188, B211, B184, B224, C180, D206, E202 and F178) were obtained from the culture collection center (Buffalo Undersea Reef Research Culture Collection). Error bars, ±s.e. (*n*=3).

Strikingly, among the *Symbiodinium* strains used in our infection study, all of the small-sized strains were from clade B, and all of the medium- and large-sized strains were from clade A. This observation supports the previous hypothesis that *Symbiodinium* cell size is strongly correlated with *Symbiodinium* genotype (LaJeunesse, 2001; LaJeunesse *et al.*, 2005). To further examine this postulate, we measured the cell size of 12 different clade A and 14 different clade B *Symbiodinium* strains of known subclade based on chloroplast large subunit (cp23S)-rDNA genotyping (Figure 6). The average cell size differed among *Symbiodinium* strains of the same clade: ranging from 7.9 to 10.1 μm in clade A and from 6.7 to 8.5 μm in clade B. Furthermore, the cell size significantly differed among *Symbiodinium* strains of the same subclade (cp23S-rDNA genotype); for instance, within the A194 subclade five phylotypes were measured with sizes ranging from 7.9 to 10.1 μm. Overall, however, clade A *Symbiodinium* strains were generally larger than clade B *Symbiodinium* strains (Figure 6). Thus our results suggest that the infectivity of different *Symbiodinium* strains can be inferred from their genotype. This hypothesis is compatible with previous results showing that clade B (type B1) *Symbiodinium* infect diverse coral species (LaJeunesse, 2002), whereas clade A (type A2) *Symbiodinium* have a high selectivity toward host species (LaJeunesse, 2001). Average cell size also varied between *Symbiodinium* strains from other clades (Figure 6), but their infectivity potential remains to be tested.

## Discussion

Symbiont specificity for a host has been proposed to be determined by the combination of cell-surface proteins on both the host cnidarian and the symbiont *Symbiodinium* during phagocytosis. However, in the present study, we found the infectivity of *Symbiodinium* strains toward both *Aiptasia* (Figure 2) and *A. tenuis* (Figure 5a) correlated with symbiont cell size. Furthermore, experiments testing the incorporation of microspheres into host cells confirmed that the size of the particle influences uptake, while also suggesting that the presence of specific cell surface proteins may not be necessary for this process (Figure 3). We therefore conclude that the infectivity potential of *Symbiodinium* strains is influenced by their cell size. This result is consistent with our general understanding of the mechanism of phagocytosis, which is known to be affected by the size of the material to be engulfed (Tabata and Ikada, 1988; Champion *et al.*, 2008). However, we still cannot exclude that large-sized *Symbiodinium* cells may be less well circulated throughout the gastrovascular cavity and therefore have a lower chance of being taken up by the host cells. It should be noted that, in our study, *Symbiodinium* CCMP2470 could quickly infect *A. tenuis* but a significant number of the symbionts dissociated from the host after 35 days (Figure 5a). Furthermore, the fluorescent microspheres that were taken into host cells were all expelled in 6 days (Figure 3a). These results suggest that at least one additional mechanism (for example, membrane surface molecules, cell division or the production of energy) is important to engage and maintain a symbiotic relationship and that creating beneficial symbiosis requires more than the sole ability of entering the host cell (Davy *et al.*, 2012).The relationship between symbiont specificity for a host and symbiont cell size has been examined previously in experiments using *Aiptasia tagetes* (Schoenberg and Trench, 1980) and the upside-down jellyfish *Cassiopeia xamachana* (Fitt, 1985). In the study using *A. tagetes*, one of the smallest *Symbiodinium* strains tested failed to infect. Conversely, in the *C. xamachana* study, one of the large *Symbiodinium* strains tested succeeded in infecting. Therefore, while previous data showed a similar trend to that being reported here, a single outlier in each experiment meant there was no clear correlation between infection and size; rather it was suggested that species specificity is not related to symbiont cell size (Schoenberg and Trench, 1980). The reason why these two studies did not find a definitive correlation between infection and size is unclear, but it indicates that additional factors may also affect species specificity and infectivity.

It is conceivable that rapid procurement of new symbionts from the environment could be important for corals to adapt to environmental changes. Previous studies have demonstrated that infection speed differs among *Symbiodinium* strains (Belda-Baillie *et al.*, 2002). However, the mechanism controlling the infection speed remained unclear. In the present study, we found that infection speed was related to symbiont cell size in both sea anemone (Figure 2c) and corals (Figure 5). This finding was supported by experimental data using microspheres of different sizes; smaller microspheres were taken into host cells significantly faster than larger ones (Figures 3a and b). However, cell size might not be the only factor determining the infection speed, and it is conceivable that cell number (Colley and Trench, 1983) and cell mortality may also influence infection speed. Nevertheless, our findings suggest that corals living in environments maintaining abundant and diverse small *Symbiodinium* strains, for example, clade B *Symbiodinium*, may have a greater chance of quickly recruiting new symbionts and therefore adapting to environmental changes.

Our results demonstrated that the maximum threshold of symbiont cell size differs between the two different coral species tested (Figure 5). It is still uncertain what determines the maximum threshold of symbiont cell size in each host species. However, as the endodermal cell size vary from 10 to 25 μm in corals and other anthozoans (Davy *et al.*, 2012), the endodermal cell size in the host could be associated with the maximum threshold of symbiont cell size. We still cannot exclude that differences in the maximum threshold of symbiont cell size is related to the ability of each host to circulate large-sized *Symbiodinium* cells throughout the gastrovascular cavity.

In coral–*Symbiodinium* associations, some coral species (generalists) harbor diverse *Symbiodinium* phylotypes, whereas others (specialists) do not (LaJeunesse, 2002; Baker, 2003). Moreover, some *Symbiodinium* phylotypes (generalists) can infect diverse host species and others (specialists) do not (LaJeunesse, 2002; Baker, 2003). Our findings suggest a possible selection mechanism of infection that can explain these observations: host species with a higher maximum threshold of symbiont cell size may acquire more diverse *Symbiodinium* phylotypes, whereas host species with a lower threshold may acquire a more limited range of phylotypes (that is, small-sized *Symbiodinium* phylotypes). Furthermore, small-sized *Symbiodinium* phylotypes may infect many host species, whereas large-sized *Symbiodinium* phylotypes may exhibit a more limited host range (that is, only hosts with a higher maximum threshold for symbiont cell size). However, not all *Symbiodinium* phylotypes able to infect a host can maintain a symbiotic relationship, for instance, when the environmental conditions are not suitable. Therefore, the combinations of host and symbiont seen in the field are likely to be determined by multiple factors, rather than infectivity alone.

Biologically enriched coral reef ecosystems sustained by healthy coral–*Symbiodinium* symbiotic associations have been in decline over the past few decades due, at least partially, to thermally induced coral bleaching (Hughes *et al.*, 2003). This decline is expected to continue if global climate change and warming continues as predicted (Hughes *et al.*, 2003). The sensitivity of corals to bleaching under increased seawater temperature differs among coral species (Marshall and Baird, 2000; Loya *et al.*, 2001). However, the bleaching sensitivity is influenced by the dominant *in situ Symbiodinium* phylotype within the coral (Glynn *et al.*, 2001), suggesting that corals may at least temporarily acclimatize to a warm environment if they possess suitable (that is, heat tolerant) *Symbiodinium* phylotypes (Baker, 2001; Baker *et al.*, 2004; Lewis and Coffroth, 2004; Rowan, 2004; Berkelmans and van Oppen, 2006; Jones *et al.*, 2008; Howells *et al.*, 2012). As the diversity of symbionts available to each coral host depends on their maximum symbiont cell size threshold (Figure 5), corals with a higher maximum threshold may have more chance to associate with suitable symbionts and therefore adapt to increased seawater temperature.

## References

[bib1] Apprill AM, Gates RD. (2007). Recognizing diversity in coral symbiotic dinoflagellate communities. Mol Ecol 16: 1127–1134. 17391401 10.1111/j.1365-294X.2006.03214.x

[bib2] Baker AC. (2001). Reef corals bleach to survive change. Nature 411: 765–766. 11459046 10.1038/35081151

[bib3] Baker AC. (2003). Flexibility and specificity in coral-algal symbiosis: diversity, ecology, and biogeography of *Symbiodinium*. Annu Rev Ecol Evol Syst 34: 661–689.

[bib4] Baker AC, Starger CJ, McClanahan TR, Glynn PW. (2004). Corals' adaptive response to climate change. Nature 430: 741–741. 15306799 10.1038/430741a

[bib5] Belda-Baillie CA, Baillie BK, Maruyama T. (2002). Specificity of a model cnidarian-dinoflagellate symbiosis. Biol Bull 202: 74–85. 11842017 10.2307/1543224

[bib6] Berkelmans R, van Oppen MJH. (2006). The role of zooxanthellae in the thermal tolerance of corals: a 'nugget of hope' for coral reefs in an era of climate change. Proc R Soc B Biol Sci 273: 2305–2312. 10.1098/rspb.2006.3567 PMC1636081 16928632

[bib7] Champion JA, Walker A, Mitragotri S. (2008). Role of particle size in phagocytosis of polymeric microspheres. Pharm Res 25: 1815–1821. 18373181 10.1007/s11095-008-9562-y PMC2793372

[bib8] Colley NJ, Trench RK. (1983). Selectivity in phagocytosis and persistence of symbiotic algae by the scyphistoma stage of the jellyfish *Cassiopeia xamachana*. Proc R Soc B-Biol Sci 219: 61–82. 10.1098/rspb.1983.0059 22470960

[bib9] Davy SK, Allemand D, Weis VM. (2012). Cell biology of cnidarian-dinoflagellate symbiosis. Microbiol Mol Biol Rev 76: 229–261. 22688813 10.1128/MMBR.05014-11 PMC3372257

[bib10] Domotor SL, Delia CF. (1986). Cell-size distributions of zooxanthellae in culture and symbiosis. Biol Bull 170: 519–525.

[bib11] Fitt WK, Trench RK. (1983). The relation of diel patterns of cell division to diel patterns of motility in the symbiotic dinoflagellate *Symbiodinium* microadriaticum Freudenthal in culture. New Phytol 94: 421–432.

[bib12] Fitt WK. (1985). Effect of different strains of the zooxanthella Symbiodinium microadriaticum on growth and survival of their coelenterate and molluscan hosts, Vol. 6. In: Delesalle B, Galzin R and Salvat B (eds). Proceeding of the 5th International Coral Reef Congress; 27 May–1 June 1985; Tahiti, French Polynesia. Antenne Museum-EPHE: Moorea, French Polynesia, pp 131–136.

[bib13] Franklin EC, Stat M, Pochon X, Putnam HM, Gates RD. (2012). GeoSymbio: a hybrid, cloud-based web application of global geospatial bioinformatics and ecoinformatics for Symbiodinium-host symbioses. Mol Ecol Resour 12: 369–373. 22018222 10.1111/j.1755-0998.2011.03081.x

[bib14] Glynn PW, Maté JL, Baker AC, Calderon MO. (2001). Coral bleaching and mortality in Panama and Ecuador during the 1997-1998 El Niño-Southern oscillation event: spatial/temporal patterns and comparisons with the 1982-1983 event. Bull Mar Sci 69: 79–109.

[bib15] Hambleton EA, Guse A, Pringle JR. (2014). Similar specificities of symbiont uptake by adults and larvae in an anemone model system for coral biology. J Exp Biol 217: 1613–1619. 24526722 10.1242/jeb.095679 PMC4006589

[bib16] Howells EJ, Beltran VH, Larsen NW, Bay LK, Willis BL, van Oppen MJH. (2012). Coral thermal tolerance shaped by local adaptation of photosymbionts. Nat Clim Chang 2: 116–120.

[bib17] Hughes TP, Baird AH, Bellwood DR, Card M, Connolly SR, Folke C et al. (2003). Climate change, human impacts, and the resilience of coral reefs. Science 301: 929–933. 12920289 10.1126/science.1085046

[bib18] Iwao K, Fujisawa T, Hatta M. (2002). A cnidarian neuropeptide of the GLWamide family induces metamorphosis of reef-building corals in the genus *Acropora*. Coral Reefs 21: 127–129.

[bib19] Jones AM, Berkelmans R, van Oppen MJH, Mieog JC, Sinclair W. (2008). A community change in the algal endosymbionts of a scleractinian coral following a natural bleaching event: field evidence of acclimatization. Proc R Soc B Biol Sci 275: 1359–1365. 10.1098/rspb.2008.0069 PMC2367621 18348962

[bib20] Kinzie RA. (1974). Experimental infection of aposymbiotic gorgonian polyps with zooxanthellae. J Exp Mar Biol Ecol 15: 335–345.

[bib21] LaJeunesse TC. (2001). Investigating the biodiversity, ecology, and phylogeny of endosymbiotic dinoflagellates in the genus *Symbiodinium* using the its region: In search of a ‘species’ level marker. J Phycol 37: 866–880.

[bib22] LaJeunesse TC. (2002). Diversity and community structure of symbiotic dinoflagellates from Caribbean coral reefs. Mar Biol 141: 387–400.

[bib23] LaJeunesse TC, Lambert G, Andersen RA, Coffroth MA, Galbraith DW. (2005). *Symbiodinium* (Pyrrhophyta) genome sizes (DNA content) are smallest among dinoflagellates. J Phycol 41: 880–886.

[bib24] Lewis CL, Coffroth MA. (2004). The acquisition of exogenous algal symbionts by an octocoral after bleaching. Science 304: 1490–1492. 15178798 10.1126/science.1097323

[bib25] Lin KL, Wang JT, Fang LS. (2000). Participation of glycoproteins on zooxanthellal cell walls in the establishment of a symbiotic relationship with the sea anemone, *Aiptasia pulchella*. Zool Stud 39: 172–178.

[bib26] Little AF, van Oppen MJH, Willis BL. (2004). Flexibility in algal endosymbioses shapes growth in reef corals. Science 304: 1492–1494. 15178799 10.1126/science.1095733

[bib27] Logan DDK, LaFlamme AC, Weis VM, Davy SK. (2010). Flow-cytometric characterisation of the cell-surface glycans of symbiotic dinoflagellates (*Symbiodinium* spp.). J Phycol 46: 525–533.

[bib28] Loya Y, Sakai K, Yamazato K, Nakano Y, Sambali H, van Woesik R. (2001). Coral bleaching: the winners and the losers. Ecol Lett 4: 122–131.

[bib29] Marshall PA, Baird AH. (2000). Bleaching of corals on the Great Barrier Reef: differential susceptibilities among taxa. Coral Reefs 19: 155–163.

[bib30] Meints RH, Pardy RL. (1980). Quantitative demonstration of cell surface involvement in a plant-animal symbiosis: lectin inhibition of reassociation. J Cell Sci 43: 239–251. 7419619 10.1242/jcs.43.1.239

[bib31] Pochon X, Gates RD. (2010). A new *Symbiodinium* clade (Dinophyceae) from soritid foraminifera in Hawai'i. Mol Phylogenet Evol 56: 492–497. 20371383 10.1016/j.ympev.2010.03.040

[bib32] Rodriguez-Lanetty M, Chang SJ, Song JI. (2003). Specificity of two temperate dinoflagellate-anthozoan associations from the north-western Pacific Ocean. Mar Biol 143: 1193–1199.

[bib33] Rowan R. (2004). Thermal adaptation in reef coral symbionts. Nature 430: 742–742. 15306800 10.1038/430742a

[bib34] Santos SR, Taylor DJ, Coffroth MA. (2001). Genetic comparisons of freshly isolated versus cultured symbiotic dinoflagellates: implications for extrapolating to the intact symbiosis. J Phycol 37: 900–912.

[bib35] Schoenberg DA, Trench RK. (1980). Genetic variation in *Symbiodinium* (=*Gymnodinium**microadriaticum* Freudenthal, and specificity in its symbiosis with marine invertebrates. III. Specificity and infectivity of *Symbiodinium* microadriaticum. Proc R Soc B Biol Sci 207: 445–460.

[bib36] Tabata Y, Ikada Y. (1988). Effect of the size and surface charge of polymer microspheres on their phagocytosis by macrophage. Biomaterials 9: 356–362. 3214660 10.1016/0142-9612(88)90033-6

[bib37] Takahashi S, Whitney S, Itoh S, Maruyama T, Badger M. (2008). Heat stress causes inhibition of the *de novo* synthesis of antenna proteins and photobleaching in cultured *Symbiodinium*. Proc Natl Acad Sci USA 105: 4203–4208. 18322010 10.1073/pnas.0708554105 PMC2393757

[bib38] Takahashi S, Yoshioka-Nishimura M, Nanba D, Badger MR. (2013). Thermal acclimation of the symbiotic alga *Symbiodinium* spp. alleviates photobleaching under heat stress. Plant Physiol 161: 477–485. 23170037 10.1104/pp.112.207480 PMC3532276

[bib39] Tchernov D, Gorbunov MY, de Vargas C, Yadav SN, Milligan AJ, Häggblom M et al. (2004). Membrane lipids of symbiotic algae are diagnostic of sensitivity to thermal bleaching in corals. Proc Natl Acad Sci USA 101: 13531–13535. 15340154 10.1073/pnas.0402907101 PMC518791

[bib40] Trench RK. (1979). Cell biology of plant-animal symbiosis. Annu Rev Plant Physiol Plant Molec Biol 30: 485–531.

[bib41] Weis VM, Reynolds WS, deBoer MD, Krupp DA. (2001). Host-symbiont specificity during onset of symbiosis between the dinoflagellates *Symbodinium* spp. and planula larvae of the scleractinian coral *Fungia scutaria*. Coral Reefs 20: 301–308.

[bib42] Wood-Charlson EM, Hollingsworth LL, Krupp DA, Weis VM. (2006). Lectin/glycan interactions play a role in recognition in a coral/dinoflagellate symbiosis. Cell Microbiol 8: 1985–1993. 16879456 10.1111/j.1462-5822.2006.00765.x

[bib43] Yellowlees D, Rees TAV, Leggat W. (2008). Metabolic interactions between algal symbionts and invertebrate hosts. Plant Cell Environ 31: 679–694. 18315536 10.1111/j.1365-3040.2008.01802.x

