# Supplementary material for: Acceptable symbiont cell size differs among cnidarian species and may limit symbiont diversity
Source: ISME J. 2017 Mar 21;11(7):1702–12. doi: 10.1038/ismej.2017.17 (PMC5520142; doi:10.1038/ismej.2017.17)
Supplement: Supplementary Table S1 [file ismej201717x1.pdf]

Table S1. *Symbiodinium* strains used for infection tests.

All *Symbiodinium* strains were obtained from culture collections. *Symbiodinium* strains whose genotype did not match the information provided from the culture collection center, or previous studies, were renamed. Phylotypes were assigned by comparison with the GeoSymbio ITS2 sequence database. For L2469, L830 and L1633, no clear match was found in the database. In these cases the clade assigned to the top blastn hit in Genbank is presented; these sequences are distinct from GeoSymbio cladeA3.

Strains	Genotypes	Original source of isolation	Cell size group
Mf1.05b	B1	Scleractinia coral (*Orbicella faveolata*)	small
CS-164	B1	Sea anemone (*Aiptasia tagetes*)	
CCMP2470	B1	Octocoral (*Antillogorgia bipinnata*)	
CCMP2462	B3	Jellyfish (*Dichotomia* sp.)	
CCMP2459	B2	Scleractinia coral (*Oculina diffusa*)	
CCMP2458	A1	Jellyfish (*Cassiopeia andromeda*)	Medium
CCMP2457	A3	Giant clam (*Tridacna crocea*)	
CCMP2464	A1	Jellyfish (*Cassiopeia xamachana*)	
CCMP2467	A1	Scleractinia coral (*Stylophora pistillata*)	
CCMP2465	A3	Giant Clam (*Tridacna maxima*)	
M2456 (*CCMP2456)	A3 (#A4)	Octocoral (*Plexaura homamalla*)	
Zs-H412	A2	Zoanthid (*Zoanthus sociatus*)	Large
L2469 (*CCMP2469)	A3 (#A1.1)	Sea anemone (*Condylactis gigantea*)	
L830 (*CCMP830)	A3 (#B)	Sea anemone (*Aiptasia pallida*)	
L1633 (*CCMP1633)	A3 (#B)	Sea anemone (*Aiptasia pulchella*)	

*, original strains: #, genetic background provided from culture collection center or previous study.
