# Supplementary material for: Acceptable symbiont cell size differs among cnidarian species and may limit symbiont diversity
Source: ISME J. 2017 Mar 21;11(7):1702–12. doi: 10.1038/ismej.2017.17 (PMC5520142; doi:10.1038/ismej.2017.17)
Supplement: Supplementary Figure S1 [file ismej201717x2.pdf]

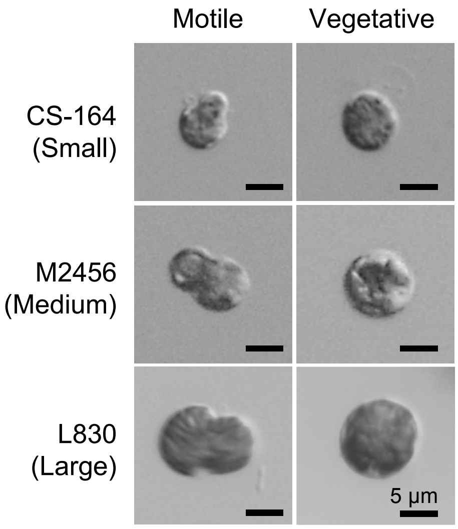

Figure S1 Cell size difference among *Symbiodinium* strains. Bright-field micrographs of cultured *Symbiodinium* CS-164 (small), M2456 (medium), and L830 (large). Left panels show motile phase cell with flagella. Right panels show vegetative phase cells.
