# Supplementary material for: Acceptable symbiont cell size differs among cnidarian species and may limit symbiont diversity
Source: ISME J. 2017 Mar 21;11(7):1702–12. doi: 10.1038/ismej.2017.17 (PMC5520142; doi:10.1038/ismej.2017.17)
Supplement: Supplementary Figure S2 [file ismej201717x3.pdf]

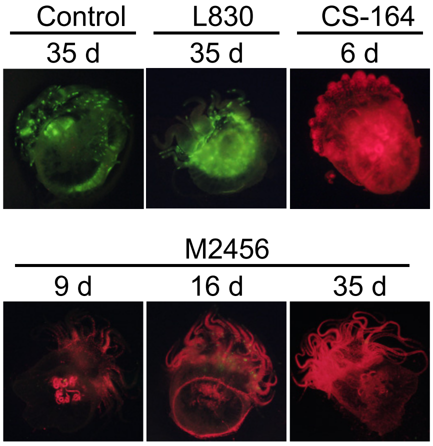

Figure S2 The potential of *Symbiodinium* to infect aposymbiotic *Aiptasia* varies among strains. Fluorescence micrographs of anemones incubated with *Symbiodinium* L830 (large), CS-164 (small), and M2456 (medium) or control (without *Symbiodinium*) after 6, 9, 16, or 35 days. d, days.
