# Supplementary material for: Acceptable symbiont cell size differs among cnidarian species and may limit symbiont diversity
Source: ISME J. 2017 Mar 21;11(7):1702–12. doi: 10.1038/ismej.2017.17 (PMC5520142; doi:10.1038/ismej.2017.17)
Supplement: Supplementary Figure S3 [file ismej201717x4.pdf]

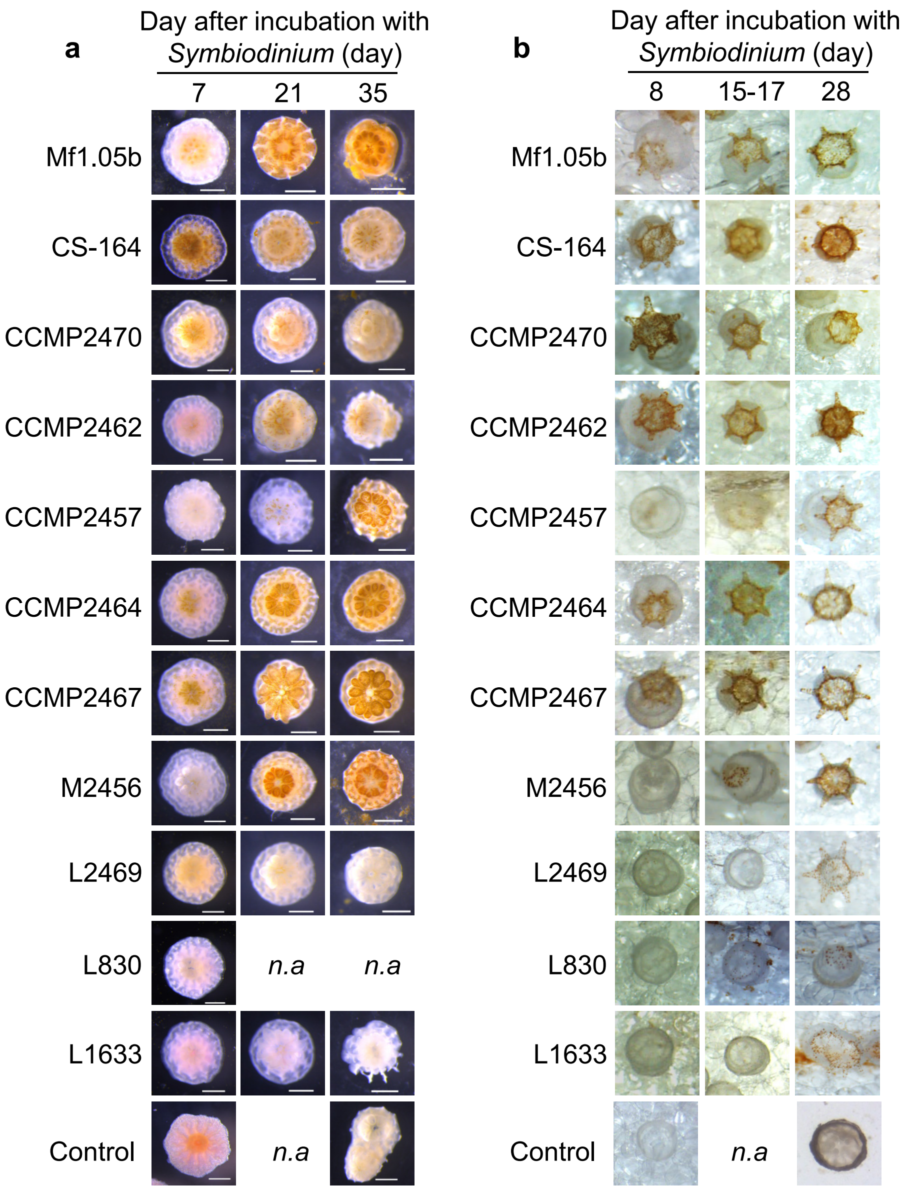

Figure S3 Infection of different *Symbiodinium* strains into corals. Aposymbiotic primal polyps of *A. tenuis* (a; scale bars, 500 µm) and *C. serailia* (b) were separately incubated with different *Symbiodinium* strains. Uptake of *Symbiodinium* into coral polyps was monitored using a stereomicroscope. Control, without *Symbiodinium*; n.a., not available.
