# Supplementary material for: Acceptable symbiont cell size differs among cnidarian species and may limit symbiont diversity
Source: ISME J. 2017 Mar 21;11(7):1702–12. doi: 10.1038/ismej.2017.17 (PMC5520142; doi:10.1038/ismej.2017.17)
Supplement: Supplementary Figure S4 [file ismej201717x5.pdf]

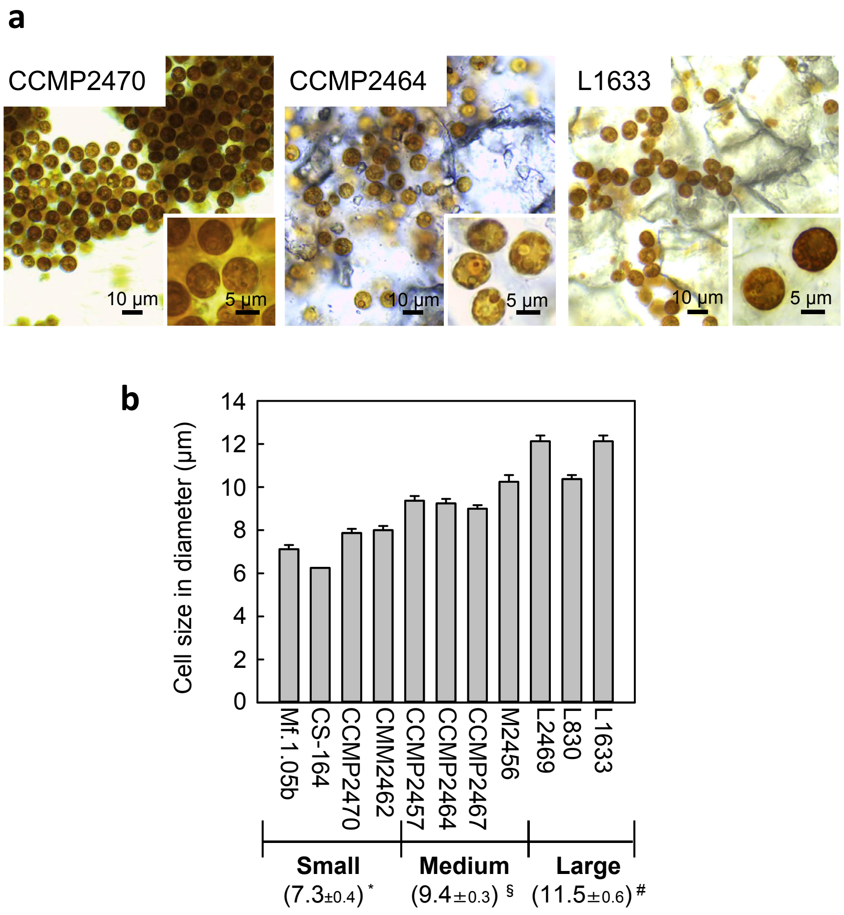

Figure S4 *Symbiodinium* cell size in corals. *Symbiodinium* cells were isolated from *C. serailia* polyps infected by different *Symbiodinium* strains. (a) *Symbiodinium* cells isolated. (b) Average cell size in diameter. Numbers below the *Symbiodinium* cell size group show the average cell diameter (µm) for all strains in that group. Different symbols (*, §, and #) indicate significant difference ($P < 0.01$) between groups. Error bars, ± standard error ($n = 10$).
